# Alcohol-related brief intervention in patients treated for opiate or cocaine dependence: a randomized controlled study

**DOI:** 10.1186/1747-597X-6-22

**Published:** 2011-08-17

**Authors:** Nelson Feldman, Anne Chatton, Riaz Khan, Yasser Khazaal, Daniele Zullino

**Affiliations:** 1 Geneva University Hospitals, Geneva, Switzerland

**Keywords:** AUDIT, brief intervention, alcohol, methadone, substance abuse

## Abstract

**Background:**

Despite the importance of heavy drinking and alcohol dependence among patients with opiate and cocaine dependence, few studies have evaluated specific interventions within this group. The aim of the present study was to evaluate the impact of screening with the Alcohol Use Disorders Identification Test (AUDIT) and of brief intervention (BI) on alcohol use in a sample of patients treated for opioid or cocaine dependence in a specialized outpatient clinic.

**Methods:**

Adult outpatients treated for opioid or cocaine dependence in Switzerland were screened for excessive alcohol drinking and dependence with the AUDIT. Patients with AUDIT scores that indicated excessive drinking or dependence were randomized into two groups--treatment as usual or treatment as usual together with BI--and assessed at 3 months and 9 months.

**Results:**

Findings revealed a high rate (44%) of problematic alcohol use (excessive drinking and dependence) among patients with opiate and cocaine dependence. The number of drinks per week decreased significantly between T0 (inclusion) and T3 (month 3). A decrease in average AUDIT scores was observed between T0 and T3 and between T0 and T9 (month 9). No statistically significant difference between treatment groups was observed.

**Conclusions:**

In a substance abuse specialized setting, screening for alcohol use with the AUDIT, followed by feedback on the score, and use of alcohol BI are both possibly useful strategies to induce changes in problematic alcohol use. Definitive conclusions cannot, however, be drawn from the study because of limitations such as lack of a naturalistic group. An important result of the study is the excellent internal consistency of AUDIT in a population treated for opiate or cocaine dependence.

## Introduction

Alcohol misuse and dependence is a major problem among opioid- and cocaine-dependent patients [1-4]. This association is linked to lower response to methadone substitution [3-5], more frequent overdose [6-9], lower quality of life [10], and higher risk of impulsive behaviors [11]. Alcohol consumption is common among cocaine users [12]. It can trigger cravings for cocaine and thus relapse [12,13] and may reduce anxiety and dysphoria induced by cocaine withdrawal [12,13], leading to alcohol use. This clinical evidence increases interest in specific alcohol interventions for patients with cocaine dependence. Despite the importance of alcohol-related problem among patients with opiate and cocaine dependence, however, few studies have evaluated specific interventions within this group.

Brief intervention (BI) is one such intervention that could be of particular interest for its simplicity and accessibility. Among excessive drinkers who were treated in primary care settings, BI reduced the percentage of excessive drinking, the quantity and frequency of alcohol consumption, and the negative consequences associated with alcohol use [14-16]. These results were confirmed in settings such as emergency services [17] and practitioners' clinics [18], as well as in various adolescent populations [19]. In addition, positive results were published in the BRAINE study, a randomized clinical trial that evaluated BI in 197 intravenous drug users [2].

Several less favorable results have also been shown, however, in studies of alcohol-dependent patients [18,20], dual diagnosis patients [21], and patients in various other settings, such as emergency [22] and general hospitals [23]. These disparities among studies are possibly due to differences in the patient and setting characteristics.

Although the efficacy of BI in different settings remains controversial, it seems to be a simple and relatively efficient intervention [24,25]. The aim of the present randomized controlled study was to evaluate the impact of screening with the Alcohol Use Disorders Identification Test (AUDIT)[26]--a 10-item self-assessment questionnaire for alcohol abuse and dependence--and of BI on alcohol use among patients treated for opioid and cocaine dependence in a specialized outpatient clinic.

## Methods

### Participants

The study was carried out in the outpatient clinic of the division of substance abuse of the University Hospitals of Geneva. For 1 year, participation in the study was proposed systematically to each adult (age ≥ 18 years) outpatient who was treated for opioid or cocaine dependence. Patients were excluded if they had an acute psychotic or manic episode, had a severe major depressive episode, patients did not understand the French language, were unable to give informed consent, or were already in treatment for problems related to alcohol misuse at the time of inclusion.

The study protocol was approved by the Geneva Ethics Committee. All participants received written information about the study and gave written informed consent.

### Measures

The participants were assessed with the following instruments:

1. Alcohol Use Disorders Identification Test (AUDIT)

The AUDIT [26] is a 10-item self-assessment questionnaire that presents good sensitivity and specificity for the screening of excessive alcohol use and dependence [26-28]. The first three questions of the AUDIT assess frequency and quantity of alcohol use. AUDIT has been translated and validated in the French language [29]. According to the cutoff values in the French validation, three groups can be identified:

(a) abstinent patients or occasional nonproblematic drinkers (score < 7 for men and < 6 for women); (b) excessive drinkers (7 ≤AUDIT score < 13 for men and 6 ≤AUDIT score < 13 for women); and (c) alcohol dependents (score > 13).

All screened patients received feedback that explained the meaning of their AUDIT score. All study participants were assessed with the AUDIT questionnaire at inclusion and those with excessive drinking or alcohol dependence were also assessed at 3 and 9 months. Diagnoses were established according to the criteria of the *International Statistical Classification of Diseases and Related Health Problems*, 10th revision (ICD-10) [30] by a resident and a senior psychiatrist.

2. Demographic and clinical characteristics collected at baseline

### Treatment allocation

Patients identified as having excessive alcohol use or dependence (AUDIT scores ≥ 6 for women or ≥ 7 for men) were randomized to receive treatment as usual (control group) or treatment as usual plus BI (intervention group). The patients in both groups were already in treatment for opioid or cocaine dependence before study inclusion. The patients allocated to BI received this intervention 2 or 3 weeks after AUDIT screening (time to assign BI to the staff after randomization and to give the patient an appointment).

1. Brief intervention

A BI was dispensed between 1 and 3 weeks after the screening with the AUDIT questionnaire by specifically trained staff. The form in which BI was dispensed was similar to that described elsewhere [14,25] and was based on the following principles and actions:

• Provide feedback to the patient about the result of the AUDIT questionnaire.

• Identify risks and discuss consequences.

• Display an emphatic and nonjudgmental attitude.

• Solicit the patient's commitment.

• Identify the goal: reduced drinking or abstinence.

• Propose a decrease in alcohol consumption with a choice of personal strategies.

• Emphasize personal responsibility for change and stimulate an attitude of change.

• Give advice and encouragement.

Promote self-observation in the consumption of alcohol.

BI was provided by a multidisciplinary team (psychiatrists, psychologists, nurses, and social workers) in the Division of Substance Abuse of the University Hospitals of Geneva. BI took 16 min with a standard deviation of 4.7. Training was provided during two workshops (4 h) by an expert in the field. He provided the staff with guidelines and information about the principles of BI.

2. Control group

The control group received treatment as usual in addition to AUDIT and score feedback. Treatment as usual refers to outpatient pharmacological and psychosocial treatment in the Division of Substance Abuse of the University Hospitals of Geneva. The outpatient staff is a multidisciplinary team: a psychiatrist, general practitioner, psychologist, nurse, and social worker.

Maintenance treatment with methadone or heroin includes medical and psychiatric follow-up, primary health care, psychosocial interventions, and administration of opiate treatments in a clinical setting. Psychosocial treatment includes medical and psychiatric follow-up, primary health care, psychosocial interventions, and, if necessary, administration of pharmacotherapy in a clinical setting.

### Outcomes

The outcomes were as follows: (a) the AUDIT scores; (b) the number of glasses of alcohol per week (1 glass: 10 g of alcohol; wine = 100 ml; beer = 250 ml; spirits = 25 ml); and (c) frequency of alcohol use (*consumption rate*). These outcomes were assessed at baseline and then at months 3 and 9.

### Randomization

Two hundred and fifty-four patients met the study inclusion criteria and accepted the invitation to participate. Of this number, and after an AUDIT screening conducted by a physician or a nurse, 112 patients were subsequently randomly assigned to intervention or control groups in a 1:1 ratio. The randomization scheme was drawn by a statistician, who used the Web site [http://www.randomizer.org/]. A random permuted block method was used, with blocks of 4 patients. The sequence was concealed from all investigators with numbered opaque sealed envelopes prepared by the statistician and handed over to the physician in charge of the study.

### Analyses

Statistical analysis was performed by using SPSS for Windows (version 18.0, IBM, Chicago, IL, USA). An initial exploratory analysis involved the calculation of proportions, means, and standard deviation to describe the baseline characteristics.

To analyze differences between participants who gave follow-up data and those who did not, we performed two logistic regressions. A binary dependent variable was generated, taking on a value of 0 if audit scores were missing at T3 and a value of 1 if audit scores were present. Type of drinkers (excessive drinkers vs. alcohol dependent) and treatment groups (treatment as usual vs. treatment as usual plus BI) served as independent variables. The same was done at T9. AUDIT internal consistency was explored by using the Cronbach α coefficient. This index varies between 0 and 1 and translates a greater degree of internal coherence if its value is close to 1. It is generally accepted that the internal consistency of an instrument is satisfactory when the value of the coefficient is equal to or above 0.70.

The evolution of AUDIT scores was analyzed by repeated measures analysis of variance (ANOVA), with treatment group (treatment as usual vs. treatment as usual plus BI), type of drinker (excessive drinker vs. alcohol dependent), and sex (male vs. female) as factors. The evolution of the quantity of alcohol consumed (number of glasses of alcohol per week) was also considered by ANOVA models, again with treatment group, type of drinker, and sex as factors. The variable that measured the quantity of alcohol consumed had to be log-transformed [x' = log(x+1)] beforehand, with the aim of making the distribution less skewed; two unlikely values, one in each group (200 glasses/week for one participant of the control group and 196 glasses/week for another of the BI group, respectively) were not considered in the analysis. In these ANOVA models, main effects, factor *× *time and factor *× *factor *× *time interactions, were paid due attention.

The consumption rate was estimated by using the first item of the AUDIT questionnaire (How often do you have a drink containing alcohol? Never (0), Monthly or less (1), Two to four times a month (2), Two to three times per week (3), Four or more times a week (4)). The first question of the AUDIT explores the frequency of alcohol consumption during the last year (never, once a month, 2 to 4 times per month, 2 or 3 times per week, 4 times or more per week).

We considered the decrease of consumption between the study time periods as a success (coded 1) and an increase or no change as a failure (coded 0). This method results in three binary outcomes (0, 1) for measures at baseline (T0), month 3 (T3), and month 9 (T9). Nonparametric Cochran's Q tests were carried out to assess whether the distribution of the values is the same for the three related dichotomous variables.

All analyses were done on a modified intention-to-treat basis. Missing data were handled by multiple imputation techniques in which scale variables were modeled with linear regression and categorical variables with logistic regression. Under the assumption that data are missing completely at random, pooled estimates were calculated and the complete data set could then be analyzed by standard methods. All statistical analyses were performed with a significant threshold of α = 0.05.

## Results

**Figure 1:**
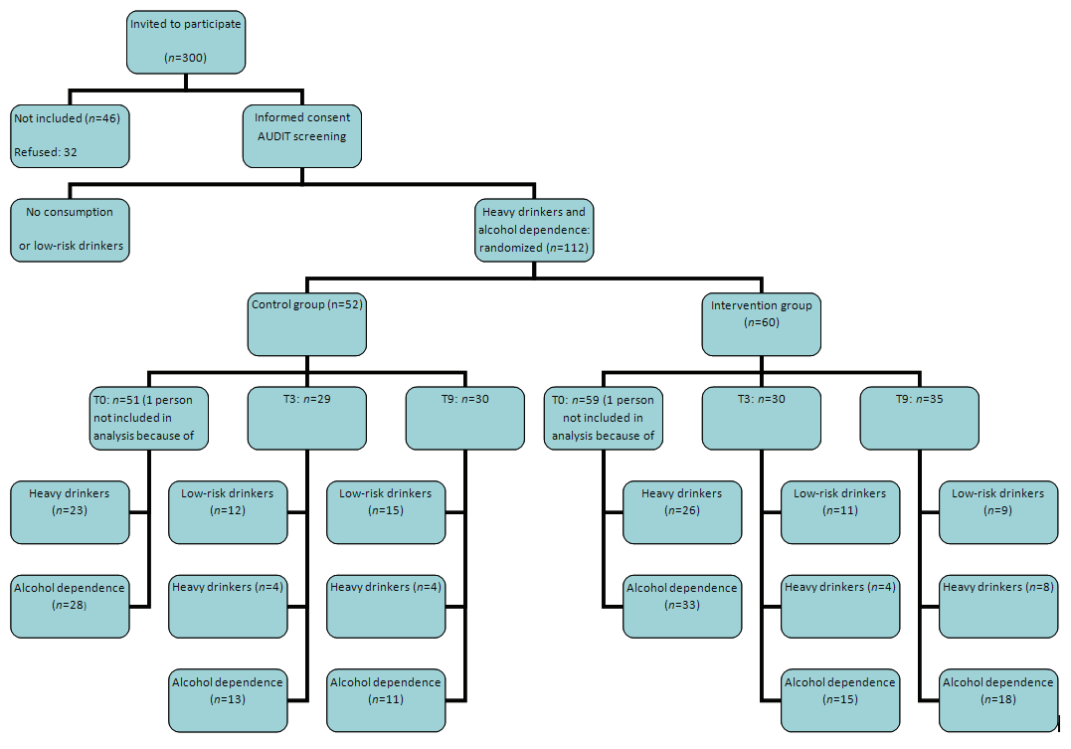
**Organigram summary of study**.

Three hundred patients were contacted for inclusion. Among them, 32 declined study participation and 14 were not included due to their engagement in an alcohol-related treatment. Finally, 254 patients (mean age: 35 years; range: 18-56; 72.3% male) signed written informed consent, completed the first evaluation, and were included in the study (Figure 1; Table 1).

**Table 1:** Baseline characteristics of the whole sample

	*N *= 254
Age [(*M *(*SD*)]	35 (7.8)
AUDIT T0 [(*M *(*SD*)]	8.8 (9)
Alcohol consumption T0 (%)	
Low-risk drinkers	55.5
Heavy drinkers	19.7
Alcohol dependence	24.8
Men (%)	72.3
Treatment (%)	
Methadone	56.2
Diacethyl-morphine (heroin)	12
Patients without substitution	31.7
Psychiatric disorder (%)	
Mood disorder	35.6
Personality disorder	34
Anxiety disorder	14.7
Psychotic disorder	9.4

AUDIT = Alcohol Use Disorders Identification Test; T0 = baseline.

Some patients were treated for opiate dependence with methadone substitution (56.2%) or diacethyl morphine (heroin treatment; 12%). Other patients had no opioid substitution and were treated for opiate or cocaine dependence (31.7%). Acceptance to participate in the study was similar (broad; wide) for both groups (patients with or without substitution). Most patients with cocaine dependence or with opiate dependence also had tobacco or cannabis dependence. Most patients had one or more concomitant psychiatric disorders (mood disorder, 35.6%; personality disorder, 34%; anxiety disorders, 14.7%; psychotic disorders, 9.4%). Logistic regressions showed that the type of drinker and treatment group did not explain the missingness of data. At T3, the odds ratio was 1.03 with confidence intervals of [0.46; 2.29] for type of drinker and 1.10 [0.50; 2.43] for treatment groups. At T9, the odds ratio was 0.78 with confidence intervals of [0.35; 1.75] for type of drinker and 1.22 [0.55; 2.70] for treatment groups. Hence, these variables displayed no particular pattern, meaning that the data for excessive drinkers and for alcohol-dependent patients, as well as for the control group and the intervention group, were equally likely to be missing.

The AUDIT shows an excellent internal consistency in this population (Cronbach α = 0.90). According to AUDIT cutoffs, 142 patients (55.9%) had no alcohol problems, whereas 112 (44.1%) were considered problematic drinkers. At inclusion, among those with problematic alcohol consumption, 43.8% were classified as excessive drinkers and 56.2% as alcohol dependents.

These 112 persons were randomized to receive BI plus treatment as usual (intervention group) or treatment as usual (control group). Because of data-entry errors, however, data for 110 persons were considered in a modified intention-to-treat analysis. Of the BI group, 59.3% completed the last observation and of the control group, 58.8% completed it (Figure 1). No differences were observed between the two treatment groups at baseline (Table 2).

**Table 2:** Baseline characteristics of the control and intervention groups

	Control Group (*N *= 52)	Intervention Group (*N *= 60)	*p *Value
Age [(*M *(*SD*)]	34.8 (6.9)	34.2 (8.6)	0.6
AUDIT T0 [(*M *(*SD*)]	16.8 (8.3)	17 (7.6)	0.9
Category of alcohol consumption: T0 %			0.8
Low-risk drinkers	0	0	
Heavy drinkers	44.2	43.3	
Alcohol dependence	55.8	56.7	
Male (%)	71.2	75	0.6
Treatment (%)	60.8	40.7	0.1
Methadone	9.8	11.9	
Diacethyl morphine (heroin) Patients without opiate substitution	29.4	47.5	

AUDIT = Alcohol Use Disorders Identification Test; T0 = baseline.

For AUDIT scores, repeated measures ANOVA shows a statistically significant overall time effect (*F*(2, 101) = 11.1 and *p *< 0.0005) and type of drinker × time interaction (*F*(2, 101) = 13.8 and *p *< 0.0005). The test of within-subject contrasts shows a significant AUDIT scores difference, irrespective of the treatment groups, between T0 and T3 (*F*(1, 102) = 16.6 and *p *< 0.0005), but not between T3 and T9. This difference reflects a decrease of the average score (Table 3). The test of within-subject contrasts also shows that AUDIT scores for excessive drinkers and alcohol dependents depend upon time and that a significant difference exists between T0 and T3 (*F*(1, 102) = 24.8 and *p *< 0.0005). During this time interval, we observed an AUDIT score reduction for alcohol-dependent patients and an increase for excessive drinkers (Table 4). As shown in Table 4, however, the mean AUDIT score for excessive drinkers remained lower than 13, the cutoff criteria for alcohol dependence. We did not observe treatment group × time nor sex × time interactions. However, there was a main effect for sex (*F*(1, 102) = 5.5 and *p *= 0.02) and for type of drinker (*F*(1, 102) = 47.8 and *p *< 0.0005), signifying that AUDIT scores depend on sex and type of drinker (Tables 4 and 5).

**Table 3:** AUDIT scores and number of alcohol drinks per week

	Control Group			Intervention Group			Control + Intervention		
	
	T0	T3	T9	T0	T3	T9	T0	T3	T9
AUDIT [*M *(*SD*)]	16.6 (8.2)	14.8 (8.9)	12.3 (8.6)	16.9 (7.7)	14.9 (7.2)	13.8 (8.7)	16.8 (7.9)	14.9 (8)	13 (8.6)
Number of drinks/week [(*M *(*SD*)]	20.9 (15)	13 (19.5)	16.4 (20.7)	25 (19.6)	15.4 (17.6)	14.7 (17.5)	23.4 (17.9)	14.5 (18.3)	15.6 (19)

AUDIT = Alcohol Use Disorders Identification Test; T0 = baseline; T3 = month 3; T9 = month 9.

**Table 4:** AUDIT scores and number of alcohol drinks per week in excessive drinkers and alcohol dependents

	Excessive Drinkers			Alcohol Dependents		
	
	T0	T3	T9	T0	T3	T9
Number of drinks per week [(*M *(*SD*)]	17.1 (12.4)	14.2 (8.1)	10.8 (8.8)	34.5 (17.8	24.8 (15.8)	24 (19.6)
AUDIT [(*M *(*SD*)]	9.4 (1.9)	11.2 (6.6)	8 (5.4)	21.9 (6.2)	17.4 (7.9)	16.6 (8.7)

AUDIT = Alcohol Use Disorders Identification Test; T0 = baseline; T3 = month 3; T9 = month 9.

**Table 5:** AUDIT scores (Mean (SD)) by group and sex

	Control Group			Intervention Group			Control + Intervention		
	
	T0	T3	T9	T0	T3	T9	T0	T3	T9
Men	17.6 (9)	16.5 (8.7)	13.9 (8.5)	17.3 (7.9)	15.1 (7)	14.4 (9.3)	17.4 (8.4)	15.7 (7.8)	14.2 (8.9)
Women	14.2 (5.4)	10.7 (8.2)	8.5 (7.8)	16 (6.9)	14.3 (8)	12.2 (6.4)	15.1 (6.2)	12.5 (8.2)	10.3 (7.2)

AUDIT = Alcohol Use Disorders Identification Test; T0 = baseline; T3 = month 3; T9 = month 9.

The ANOVA for the quantity of alcohol consumed, with treatment group, type of drinker, and sex as factors, shows an effect of time that is statistically significant (*F*(2, 101) = 15.7 and *p *< .0005). The contrast values give a statistically significant difference toward a decrease between T0 and T3 (*F*(1, 102) = 29.6 and *p *< .0005). No factor × time interactions were observed. Nevertheless, a main effect was observed for type of drinker (*F*(1, 102) = 9 and *p *= .003), showing that a statistically significant difference exists between excessive drinkers and alcohol dependents for the overall number of alcoholic drinks consumed (Tables 4 and 6).

**Table 6:** Number of alcohol drinks per week by group and gender

	Control Group			Intervention Group			Control + Intervention		
	
	T0	T3	T9	T0	T3	T9	T0	T3	T9
Men	31 (20)	22.9 (16.4)	20.6 (17.2)	28.8 (17.6)	21 (14)	20.4 (20.6)	29.8 (18.7)	21.8 (15)	20.5 (19)
Women	22 (13.4)	14.8 (12)	12.3 (11.3)	19.7 (15.1)	18.5 (9.8)	15.1 (9.8)	20.8 (14)	16.7 (11)	13.7 (10.5)

AUDIT = Alcohol Use Disorders Identification Test; T0 = baseline; T3 = month 3; T9 = month 9.

The main changes in alcohol consumption (AUDIT scores, number of drinks, and frequency) are observed at T3, but changes in frequency and number of drinks do not persist at T9. To check whether the rates of alcohol consumption (according to the first AUDIT question) differ across time for the intervention and control groups, we used the Cochran Q test. This test did not find a significant statistical distribution difference across time (Table 7), either for the patients benefitting from the BI (*Q *= 0.5 and *p *= 0.6), or for the control group (*Q ***= 1**.1 and *p *= 0.4).

**Table 7:** Evolution of alcohol consumption by treatment group

	Control Group		Intervention Group	
	
	T0-T3	T3-T9	T0-T3	T3-T9
Increased or unchanged alcohol use: failure (*n*)	16	14	46	52
Decreased alcohol use: success (*n*)	35	37	13	7

AUDIT = Alcohol Use Disorders Identification Test; T0 = baseline; T3 = month 3; T9 = month 9.

## Discussion

In the present study, significant decreases in AUDIT scores were observed at T3 and T9 in both treatment groups, without gender differences, as previously found elsewhere [14,18,20,31]. In our study, gender differences were not linked to treatment effect but to a more general pattern of alcohol consumption. Similarly, a significant decrease in alcohol consumption was observed at T3. The main changes in alcohol consumption (AUDIT scores, number of drinks, and frequency) are observed at T3, but changes in frequency and number of drinks do not persist at T9. This is possibly due to a reduction of the intervention effect over time.

Of interest, these results are in accordance with other studies founding a significant decrease in the consumption of alcohol following BI [14,15], including among a population of intravenous drug users [2]. Astonishingly, dependent subjects improved better than excessive drinkers in AUDIT scores between T0 and T3. This difference between types of drinker was not observed for the number of drinks consumed per week. In the present setting, BI did not have an additional effect in comparison with AUDIT screening. This is possibly due to a lower impact of BI in this target population [20,21,32]. BI has previously been found to be less efficient among alcohol dependents [14,24]. Furthermore, lack of differences between interventions may simply show that screening alcohol use with AUDIT and providing feedback about the score has a positive impact on alcohol-drinking behaviors [33]. This phenomenon has been evoked by different studies and could correspond to what Jenkins et al. call reactivity to assessment [34]. The results were also possibly due to spontaneous adaptations of the naturalistic "treatment as usual" received by both groups following AUDIT screening or BI.

In this study, screening with the AUDIT, as well as the BI, induced changes in alcohol consumption among heroin and cocaine users in treatment. This result possibly means that in an addiction clinic, both strategies were useful. In the absence of a naturalistic or waiting list group, we cannot fully exclude a spontaneous favorable evolution linked to time or to other concomitant factors such as treatment as usual. It is probable, however, that in the absence of a specific intervention targeting alcohol consumption, patients treated for opiate or cocaine addiction may not usually receive sufficient attention for alcohol misuse.

In fact, one of the limitations of the present study is the lack of data about naturalistic change during treatment as usual. Participation in the study was voluntary and all participants were selected by convenience sampling when they came to the Division of Substance Abuse for treatment. As there is a lack of studies that have evaluated the longitudinal effect of the intervention within this specific group of drug-addicted patients, no a priori sample size calculation had been undertaken. Indeed, the extreme heterogeneity of patient characteristics, health care, and study settings made it difficult to provide reliable effect size estimates for sample size calculation for continuous endpoints. If we had to take, for instance, an effect size of 30% and a correlation among the repeated observations of 0.4, using the equation for sample size calculations given by Diggle et al. [35], with a power of 0.8, would yield a minimum sample size of 82 persons in each arm. This estimate, if a plausible guess, shows that the current study sample size may have proven insufficient to reject the null hypothesis at the 0.05 level. Hence, the study may be underpowered and may have led to discarding the BI as a potentially useful treatment.

Nevertheless, because of the simplicity of the proposed interventions and the relatively important improvements observed (reduction in the number of drinks per week from 20.9 at T0 to 13 at T3 in the control group and from 25 at T0 to 15.4 at T3 in the intervention group), systematic screening with AUDIT, followed by feedback on the score, and/or BI for alcohol misuse among outpatients treated for opiate or cocaine dependence will be promoted and investigated as having promising potential in this target group. The screening, followed by feedback on the problematic use of alcohol, is an interesting strategy to include in a population of heroin and cocaine users. New tools, such as the Alcohol, Smoking and Substance Involvement Screening Test (ASSIST) questionnaire, deserve to be evaluated in future research for screening the use of alcohol and other substances [36].

As previously found [1], another important result of the present study is the excellent internal consistency of AUDIT in a population of patients treated for opiate or cocaine dependence. Using this instrument, we found a 44% rate for problematic alcohol consumption (excessive drinking and dependence), confirming results of other studies on similar populations [2,3].

## Competing interests

The authors declare that they have no competing interests.

## Authors' contributions

NF designed the study. AC conducted the statistical analyses. NF, AC, and YK wrote the first draft and compiled the co-authors' suggestions. All authors participated in the drafting of the manuscript and approved the final version.
