# Correction: Alcohol-related brief intervention in patients treated for opiate or cocaine dependence: a randomized controlled study

**DOI:** 10.1186/1747-597X-8-28

**Published:** 2013-08-01

**Authors:** Nelson Feldman, Anne Chatton, Riaz Khan, Yasser Khazaal, Daniele Zullino

**Affiliations:** 1 Geneva University Hospitals, Geneva, Switzerland

## Correction

After publication of this article, it came to our attention that the published manuscript contained errors in the table three (Table 1), Table four (Table 2), Table five (Table 3) and Table six (Table 4), Table seven (Table 5). The original published article contains old tables from the first version of the manuscript sent for initial consideration for publication.

**Table 1:** AUDIT scores and number of weekly alcohol drinks by group

	**CONTROL GROUP**			**INTERVENTION GROUP**			**CONTROL AND INTERVENTION GROUPS**		
	**T0**	**T3**	**T9**	**T0**	**T3**	**T9**	**T0**	**T3**	**T9**
AUDIT, Mean (±SD)	16.6 (8.2)	12.4 (7.5)	11.6 (7.3)	16.9 (7.7)	13 (6.2)	13.9 (8.1)	16.8 (7.9)	12.7 (6.8)	12.8 (7.8)
Number of drinks/week, Mean (±SD)	23.8 (9.5)	16.3 (12.6)	18.7 (13.2)	25.3 (14.3)	17 (11.6)	18.4 (10.4)	24.6 (12.3)	16.7 (12)	18.5 (11.7)

**Table 2:** AUDIT scores and number of alcohol drinks per week in excessive drinkers and alcohol dependents

	**EXCESSIVE DRINKERS**			**ALCOHOL DEPENDENTS**		
	**T0**	**T3**	**T9**	**T0**	**T3**	**T9**
AUDIT, Mean (± SD)	9.4 (1.2)	10.6 (6)	9.4 (5.8)	21.9 (6.2)	14.2 (7)	15.2 (8.1)
Number of drinks per week, Mean (± SD)	19.4 (9.2)	13.4 (7)	15.1 (8.3)	28.2 (12.9)	19 (14.1)	20.9 (13.1)

**Table 3:** AUDIT scores according to group and gender

	**CONTROL GROUP**			**INTERVENTION GROUP**			**CONTROL AND INTERVENTION GROUPS**		
	**T0**	**T3**	**T9**	**T0**	**T3**	**T9**	**T0**	**T3**	**T9**
Men	17.6 (9)	13.4 (7.4)	13.4 (6.8)	17.3 (7.9)	13.5 (6.3)	14.3 (8.7)	17.4 (8.4)	13.4 (6.8)	13.6 (8)
Women	14.2 (5.4)	10 (7.4)	9 (7.3)	16 (6.9)	11.3 (5.7)	12.6 (6)	15.1 (6.2)	10.7 (6.5)	10.8 (6.8)

**Table 4:** Number of alcohol drinks per week according to group and gender

	**CONTROL GROUP**			**INTERVENTION GROUP**			**CONTROL AND INTERVENTION GROUPS**		
	**T0**	**T3**	**T9**	**T0**	**T3**	**T9**	**T0**	**T3**	**T9**
Men	23.9 (10.3)	17.7 (13.6)	19.8 (14.4)	26.7 (15.3)	17.7 (12.9)	18.5 (11)	25.5 (13.3)	17.7 (13.1)	19.1 (12.5)
Women	23.4 (7.7)	13.1 (9.3)	16 (9.9)	21.2 (10.1)	14.9 (6.5)	18 (8.8)	22.3 (8.9)	14 (7.9)	17 (9.3)

**Table 5:** Evolution of frequency of alcohol consumption by treatment group

	**CONTROL GROUP**			**INTERVENTION GROUP**		
	**T0-T3**	**T3-T9**	**T0-T9**	**T0-T3**	**T3-T9**	**T0-T9**
Increased or unchanged frequency of alcohol use: % failure (n)	68.6 (35)	78.4 (40)	70.6 (36)	64.4 (38)	71.2 (42)	67.8 (40)
Decreased frequency of alcohol use: % success (n)	31.4 (16)	21.6 (11)	29.4 (15)	35.6 (21)	28.8 (17)	32.2 (19)

This correction article contains the tables revised after peer review of the manuscript.

The authors and Publisher apologize to the readers for the inconvenience caused.

This correction article contains the revised tables from the original article [1] Tables one and two remain unchanged.
